# Patient-Reported Financial Burden of Treatment for Colon or Rectal Cancer

**DOI:** 10.1001/jamanetworkopen.2023.50844

**Published:** 2024-01-09

**Authors:** Sheetal Kircher, Fenghai Duan, Na An, Ilanan F. Gareen, JoRean D. Sicks, Gelareh Sadigh, Jennifer M. Suga, Heather Kehn, Paul T. Mehan, Rajesh Bajaj, David S. Hanson, Samir M. Dalia, Jared D. Acoba, Demet Gokalp Yasar, Elyse R. Park, Lynne I. Wagner, Ruth C. Carlos

**Affiliations:** 1 Robert H. Lurie Comprehensive Cancer Center of Northwestern University, Chicago, Illinois; 2 Department of Biostatistics, Brown University School of Public Health, Providence, Rhode Island; 3 Center for Statistical Sciences, Brown University School of Public Health, Providence, Rhode Island; 4 Department of Epidemiology, Brown University School of Public Health, Providence, Rhode Island; 5 University of California School of Medicine, Irvine; 6 Kaiser Permanente NCI Community Oncology Research Program and NCORP, Vallejo, California; 7 Metro-Minnesota Community Oncology Research Consortium, St Louis Park; 8 Missouri Baptist Hospital NCORP, Saint Louis; 9 Carolina Health Care and NCORP, Florence, South Carolina; 10 Mary Bird Perkins Cancer Center, Baton Rouge, Louisiana; 11 Mercy Hospital, Joplin, Missouri; 12 University of Hawaii Cancer Center, Honolulu; 13 Marshfield Clinic, Minocqua, Wisconsin; 14 Massachusetts General Hospital Cancer Center, Boston, Massachusetts; 15 Wake Forest University Health Sciences, Winston-Salem, North Carolina; 16 University of Michigan Comprehensive Cancer Center, Ann Arbor

## Abstract

**Importance:**

The longitudinal experience of patients is critical to the development of interventions to identify and reduce financial hardship.

**Objective:**

To evaluate financial hardship over 12 months in patients with newly diagnosed colorectal cancer (CRC) undergoing curative-intent therapy.

**Design, Setting, and Participants:**

This prospective, longitudinal cohort study was conducted between May 2018 and July 2020, with time points over 12 months. Participants included patients at National Cance Institute Community Oncology Research Program sites. Eligibility criteria included age at least 18 years, newly diagnosed stage I to III CRC, not started chemotherapy and/or radiation, treated with curative intent, and able to speak English. Data were analyzed from December 2022 through April 2023.

**Main Outcomes and Measures:**

The primary end point was financial hardship, measured using the Comprehensive Score for Financial Toxicity (COST), which assesses the psychological domain of financial hardship (range, 0-44; higher score indicates better financial well-being). Participants completed 30-minute surveys (online or paper) at baseline and 3, 6, and 12 months.

**Results:**

A total of 450 participants (mean [SD] age, 61.0 [12.0] years; 240 [53.3%] male) completed the baseline survey; 33 participants (7.3%) were Black and 379 participants (84.2%) were White, and 14 participants (3.1%) identified as Hispanic or Latino and 424 participants (94.2%) identified as neither Hispanic nor Latino. There were 192 participants (42.7%) with an annual household income of $60 000 or greater. There was an improvement in financial hardship from diagnosis to 12 months of 0.3 (95% CI, 0.2 to 0.3) points per month (*P* < .001). Patients with better quality of life and greater self-efficacy had less financial toxicity. Each 1-unit increase in Functional Assessment of Cancer Therapy–General (rapid version) score was associated with an increase of 0.7 (95% CI, 0.5 to 0.9) points in COST score (*P* < .001); each 1-unit increase in self-efficacy associated with an increase of 0.6 (95% CI, 0.2 to 1.0) points in COST score (*P* = .006). Patients who lived in areas with lower neighborhood socioeconomic status had greater financial toxicity. Neighborhood deprivation index was associated with a decrease of 0.3 (95% CI, −0.5 to −0.1) points in COST score (*P* = .009).

**Conclusions and Relevance:**

These findings suggest that interventions for financial toxicity in cancer care should focus on counseling to improve self-efficacy and mitigate financial worry and screening for these interventions should include patients at higher risk of financial burden.

## Key Points

**Question:**

What is the trajectory of financial hardship experienced by patients treated for early-stage colorectal cancer during the 12 months after diagnosis?

**Findings:**

In this cohort study of 450 patients with colorectal cancer treated with curative intent in community settings, financial hardship was common at diagnosis and decreased over time, but some groups, such patients with low self-efficacy, unemployed individuals, or patients with government insurance, did not improve as much as their counterparts.

**Meaning:**

These findings suggest that interventions for patients with newly diagnosed cancer should focus on counseling to decrease financial worry and screening for these interventions should ensure inclusion of patients at greater risk for financial hardship.

## Teaser

This cohort study evaluates patient-reported financial hardship during 12 months among patients with newly diagnosed colorectal cancer undergoing curative-intent therapy.

## Introduction

The number of cancer treatment options has increased over the past 2 decades, leading to improvements in outcomes in many cancer types.^1^ However, these advancements come with a high financial cost to the system and, more concerningly, to patients. The conceptual model of financial hardship consists of 3 domains: material conditions (eg, out of pocket expense, employment limitation, debt), psychological response (eg, distress), and coping behaviors (eg, delay, forgoing care, or nonadherence due to cost).^2^

Most work in financial hardship has focused on patients with advanced cancers or used a retrospectively assembled heterogenous group, representing a knowledge gap for patients with early-stage cancer. In patients with early-stage colorectal cancer (CRC), covariates of higher burden include younger age, being uninsured or unemployed, lower income, and having received chemotherapy.^3,4,5^ Financial hardship is associated with low health literacy,^6,7^ poor quality of life,^8^ and early mortality.^9^ However, most of these studies are retrospective and cross-sectional, limiting our understanding of the onset and trajectory of financial hardship throughout the treatment continuum. The longitudinal experience of homogenous patient populations is critical so interventions, such as financial counseling, can occur in the right population at the appropriate time. We conducted a longitudinal, prospective cohort study to evaluate the 12-month trajectory of financial hardship in patients with a new diagnosis of CRC and undergoing curative-intent therapy at community oncology sites.

## Methods

### Study Design and Eligibility

This cohort study was approved by the National Cancer Institute Central Institutional Review Board, and all participants gave written informed consent. This study was a longitudinal cohort study coordinated by ECOG-ACRIN. Patients were enrolled through National Cancer Institute Community Oncology Research Program community sites. Eligibility included age at least 18 years, new diagnosis of CRC within 60 days of registration and not started chemotherapy and/or radiation, stage I to III disease treated with curative intent, and English speaking. This report follows the Strengthening the Reporting of Observational Studies in Epidemiology (STROBE) reporting guideline for cohort studies. This study was registered on ClinicalTrials.gov (NCT03516942).

### Questionnaires

Participants completed 30-minute survey instruments at baseline and 3, 6, and 12 months to assess changes in financial hardship, employment, and access to resources. The questionnaires were completed online or on paper at home or in clinic based on patients’ preference. This report focuses on the primary objective analysis to evaluate the change in self-reported financial hardship from baseline over 12 months.

Participants were asked their health insurance status, sociodemographic (age, sex, race and ethnicity, marital status, education, household income, employment) and clinical characteristics (treatment plan, comorbid conditions). Race and ethnicity were self-reported and defined according to the National Institutes of Health Policy on Reporting Race and Ethnicity Data, categorized as Black, White, or other race (including American Indian or Alaska Native, Asian, multiple races, Native Hawaiian or Other Pacific Islander, not reported, and unknown) and Hispanic or Latino, not Hispanic or Latino or unknown ethnicity.^10^ Race and ethnicity were assessed because they have been reported as factors associated with financial hardship in previous work. The Comprehensive Score for Financial Toxicity (COST)^11^ was used to measure the primary end point. COST is an 11-item patient-reported outcome (PRO) of financial hardship that uses a 7-day time window and 5-point Likert response scale (ranging from 0, indicating not at all, to 4, very much). Scoring has been previously described,^11^ and higher COST scores (range, 0-44) represent better financial well-being. The COST survey measures the psychological domain of financial hardship and represents a patient’s distress related to the cost of their cancer care. Research coordinators completed forms with health insurance status and clinical characteristics (date of diagnosis, cancer type, stage, vital status, treatment plan) at all time points.

Neighborhood Deprivation Index (NDI)^12^ is a continuous variable calculated based on neighborhood socioeconomic status (SES). Neighborhood SES uses a weighted combination of neighborhood characteristics (percentage of households with a mean of ≥1 person per room, median value of homes, percentage of households living below poverty level, median household income, percentage of individuals ≥25 years with a bachelor’s degree or higher, percentage of individuals ≥25 years with less than a 12th grade education, and the percentage of individuals ≥16 years in the labor force who are unemployed) scaled between 0 and 100, with higher number indicating greater neighborhood SES.^13^ The participant’s 5-digit zip code was mapped to county-level data^14,15^ and when a participant’s zip code represented multiple counties, aggregate means were used for county-level estimates. NDI is calculated as 100 – neighborhood SES score. Higher NDI scores are indicative of greater neighborhood deprivation, and results are divided into quartiles, with quartile 4 (the highest score) having the highest deprivation.

The Functional Assessment of Cancer Therapy–General (FACT-G) is a validated measure that incorporates symptoms and functional domains to assess quality of life,^16^ and we used the rapid version (FACT-G7) to reduce survey burden (range, 0-28; higher score indicates better quality of life).^17^ Self-efficacy was assessed with the 6-item Stanford Self-Efficacy for Management Chronic Disease (range, 1-10; higher score indicates better self-efficacy).^18^

### Statistical Analysis

#### Primary Aim

The primary aim was to evaluate the change in self-reported financial hardship of patients diagnosed with CRC treated with curative-intent from registration (within 60 days of diagnosis) to 12 months. The assessment included the analysis of longitudinal trajectory of COST over 12 months, as well as the 12-month difference (12-month score − baseline) COST.

Baseline characteristics were summarized with mean SD, median, and IQRs for the continuous variables, while frequencies and percentages were summarized for categorical variables across all time points.

We used 2 modeling techniques to investigate the association of COST between baseline and the 12-month follow-up: a longitudinal model for the cross-sectional analysis and an ordinary linear regression, referred to as the *difference model*. The longitudinal model can incorporate COST measurements from all 4 time points and indirectly derive changes in COST by investigating the time covariate. Conversely, the difference model directly investigates the change of COST but is limited to patients with both baseline and 12-month follow-up COST measurements.

A longitudinal model of COST in the linear mixed-effects regression was performed to include measurements at 4 follow-up visits (baseline and 3-, 6-, and 12-month follow-ups). The covariates included the baseline variables (such as age, sex, race, marital status, education, household income, primary insurance, employment, cancer type, stage, chemotherapy plan, comorbidities, FACT-G7, self-efficacy, NDI, and safety-net hospital), time, and chemotherapy plan that were informed by baseline and 3-month data. Except for NDI, all other covariates were protocol-specified covariates. Due to the limited number of patients identifying as Hispanic or Latino, ethnicity was excluded from the final model to avoid issues related to data sparsity. Longitudinal models used a compound-symmetry covariance structure by including a random intercept for each participant in the models. The covariates were modeled as fixed effects. The longitudinal model of COST trajectory used continuous time (in months) grouped by the 4 time points. Surveys completed too early (more than 35 days prior) or too late (more than the 95 percentiles) were excluded from the analysis. To assess the associations of the covariates with COST over time and to assess the influence of the dropout rate with the association of the covariates with COST over time, 2 additional longitudinal models were fit: a model with baseline and 3-month follow-up and a model with baseline and 3- and 6-month follow-ups. We assessed 2-way interactions between each covariate and the time prior to the main outcome modeling, and none of the interactions reached statistical significance.

In addition, we used the difference model to model the change of COST from baseline to 12-month follow-up, with the same set of baseline covariates (except time) as the longitudinal models. The COST measure at baseline was included as a covariate. All participants with measurements at both baseline and the 12-month follow-up were included in the model.

*P* values were 2-sided, and *P* = 0.05 was used to determine statistical significance. Statistical analyses were performed using SAS software version 9.4 (SAS Institute). The main findings were derived from the longitudinal model of COST, using data from all 4 follow-ups. All other models were considered supportive analyses. Moreover, in the multiple regression analysis, the significance was determined based on the overall *P* value (or type III *P* value) for categorical covariates; therefore, no correction for multiple comparisons was applied during the analyses. Data were analyzed from December 2022 through April 2023.

#### Missing Data Imputation

The missing values in categorical variables were imputed by creating a new category to represent missing values.^19^ For continuous variables, we used multiple imputation to handle missing values by generating multiple data sets.^20^ We used least absolute shrinkage and selection operator to combine the results across the imputed data sets, resulting in more robust and stable regression coefficients.^21^ The analysis following multiple imputation, termed a sensitivity analysis, was conducted using functions in the R package mice version 4.3.1 (R Project for Statistical Computing).

#### Sample Size

The study was powered by enrolling 563 patients to detect a 10% difference in COST scores from baseline to the 12-month follow-up at an α = .05. The calculation was done using PASS sample size software version 14 (NCSS).

## Results

### Patient Characteristics

**Figure 1.**
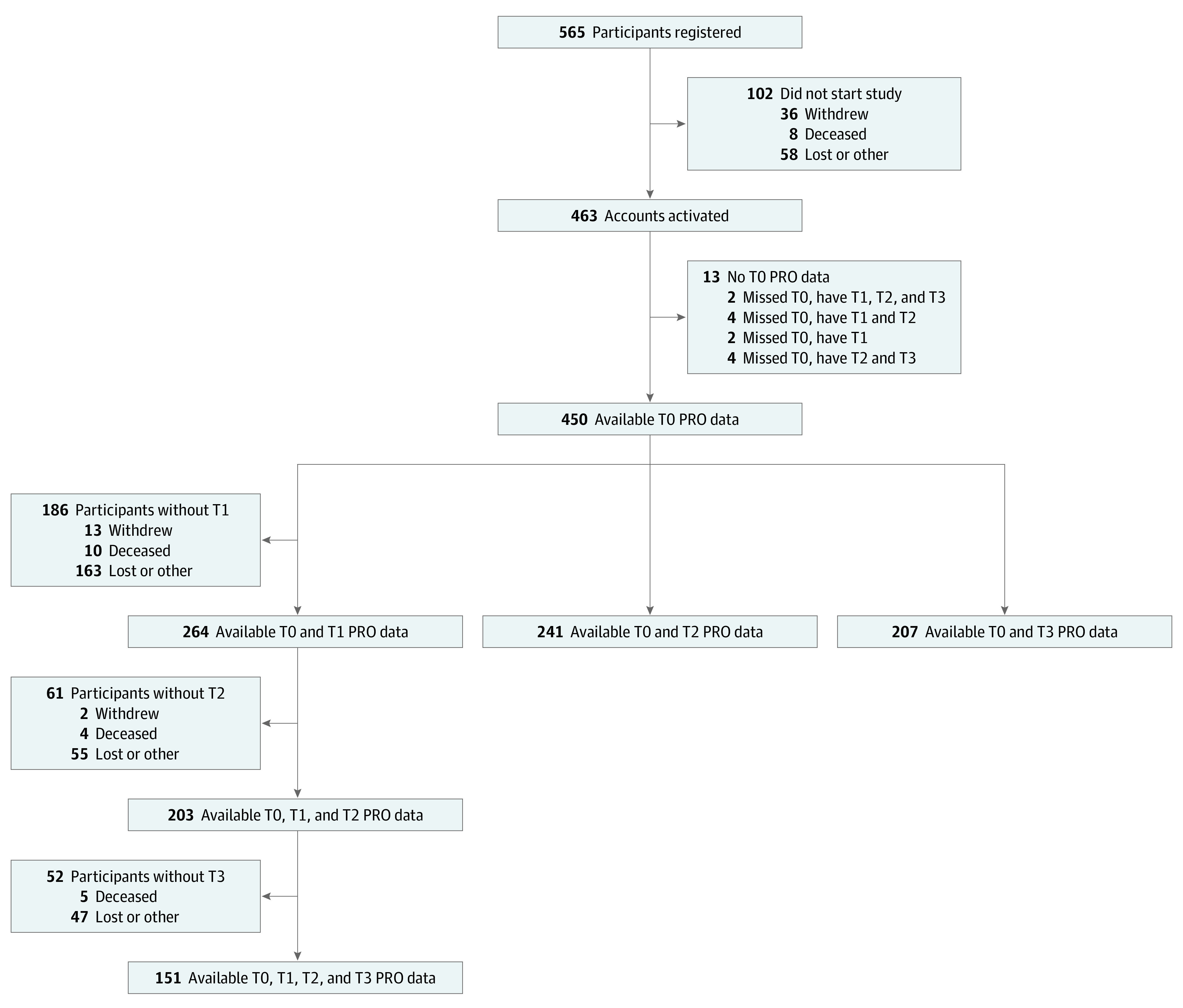
Participant Recruitment Flowchart PRO indicates patient-reported outcome; T0, baseline; T1, 3-month follow-up; T2, 6-month follow-up; and T3, 12-month follow-up.

A total of 565 patients were registered from 172 sites between May 2018 and July 2020 (Figure 1). Of 565 patients registered, 450 patients (mean [SD] age, 61.0 [12.0] years; 240 [53.3%] male) completed the baseline COST measure (Table 1). Based on the limited data from nonparticipants, we observed no difference between individuals who opted to participate in the study and those who chose not to (eTable 4 in Supplement 1). By self-reported race, there were 33 Black participants (7.3%) and 279 White participants (84.2%), and by self-reported ethnicity, there were 14 Hispanic or Latino participants (3.1%) and 424 participants (94.2%) who were not Hispanic or Latino. Most participants were married (295 participants [65.6%]), and had an annual household income of $60 000 or greater (192 participants [42.7%]). The mean (SD) FACT-G7 score was 17.9 (5.7). The other sociodemographic characteristics and PRO responses are presented in Table 1.

**Table 1.** Participant Characteristics by Follow-Up Assessment Completed

Characteristic	Participants by follow-up, No. (%)			
	Baseline (n = 450)	3-mo (n = 264)	6-mo (n = 241)	12-mo (n = 207)
COST per time point, mean (SD)	23.5 (11.9)	25.0 (12.4)	26.0 (12.1)	28.6 (11.7)
Age, mean (SD), y	61.0 (12.0)	61.6 (12.4)	62.0 (12.3)	62.0 (12.1)
Sex				
Male	240 (53.3)	138 (52.3)	125 (51.9)	108 (52.2)
Female	210 (46.7)	126 (47.7)	116 (48.1)	99 (47.8)
Race				
Black	33 (7.3)	16 (6.1)	15 (6.2)	10 (4.8)
White	379 (84.2)	232 (87.9)	210 (87.1)	186 (89.9)
Other^a^	38 (8.4)	16 (6.1)	16 (6.6)	11 (5.3)
Ethnicity				
Hispanic or Latino	14 (3.1)	8 (3.0)	6 (2.5)	2 (1.0)
Not Hispanic or Latino	424 (94.2)	249 (94.3)	228 (94.6)	198 (95.7)
Not reported	4 (0.9)	2 (0.8)	2 (0.8)	3 (1.4)
Unknown	8 (1.8)	5 (1.9)	5 (2.1)	4 (1.9)
Education				
≤High school	178 (39.6)	96 (36.4)	83 (34.4)	69 (33.3)
Some college and advanced degree	267 (59.3)	165 (62.5)	156 (64.7)	137 (66.2)
Not answered	5 (1.1)	3 (1.1)	2 (0.8)	1 (0.5)
Marital status				
Married, living with partner	295 (65.6)	176 (66.7)	168 (69.7)	134 (64.7)
Unpartnered	153 (34.0)	87 (33.0)	72 (29.9)	71 (34.3)
Not answered	2 (0.4)	1 (0.4)	1 (0.4)	2 (1.0)
Annual household income, $				
≤29 999	111 (24.7)	58 (22.0)	53 (22.0)	45 (21.7)
30 000 to 59 999	136 (30.2)	76 (28.8)	70 (29.0)	56 (27.1)
≥60 000	192 (42.7)	123 (46.6)	112 (46.5)	101 (48.8)
Not answered	11 (2.4)	7 (2.7)	6 (2.5)	5 (2.4)
Employment				
Employed	214 (47.6)	124 (47.0)	113 (46.9)	101 (48.8)
Retired	153 (34.0)	102 (38.6)	93 (38.6)	78 (37.7)
Unemployed	77 (17.1)	36 (13.6)	33 (13.7)	25 (12.1)
Not answered	6 (1.3)	2 (0.8)	2 (0.8)	3 (1.4)
Safety net hospital				
Yes	66 (14.7)	31 (11.7)	32 (13.3)	30 (14.5)
No or unknown	384 (85.3)	233 (88.3)	209 (86.7)	177 (85.5)
Chemotherapy				
Yes	267 (59.3)	158 (59.8)	136 (56.4)	113 (54.6)
No	183 (40.7)	106 (40.2)	105 (43.6)	94 (45.4)
Primary health insurance				
Private insurance	223 (49.6)	133 (50.4)	114 (47.3)	99 (47.8)
Military, Indian, or Medicare	197 (43.8)	118 (44.7)	116 (48.1)	100 (48.3)
Medicaid, single service, or no insurance	30 (6.7)	13 (4.9)	11 (4.6)	8 (3.9)
Cancer type				
Colon cancer	289 (64.2)	168 (63.6)	157 (65.1)	132 (63.8)
Rectal cancer	140 (31.1)	82 (31.1)	74 (30.7)	67 (32.4)
Rectosigmoid junction	21 (4.7)	14 (5.3)	10 (4.1)	8 (3.9)
Cancer stage				
I	68 (15.1)	40 (15.2)	45 (18.7)	39 (18.8)
II	140 (31.1)	85 (32.2)	70 (29.0)	67 (32.4)
III	242 (53.8)	139 (52.7)	126 (52.3)	101 (48.8)
Comorbidities, No.				
>1	239 (53.1)	140 (53.0)	122 (50.6)	104 (50.2)
1	111 (24.7)	65 (24.6)	64 (26.6)	56 (27.1)
None	100 (22.2)	59 (22.3)	55 (22.8)	47 (22.7)
NDI^b^				
Quartile				
≤41.9 (Lowest)	117 (26.0)	72 (27.3)	62 (25.7)	59 (28.5)
42.0 to <44.5	99 (22.0)	58 (22.0)	55 (22.8)	46 (22.2)
44.6 to 47.0	122 (27.1)	71 (26.9)	68 (28.2)	55 (26.6)
>47.0 (Highest)	112 (24.9)	63 (23.9)	56 (23.2)	47 (22.7)
Median (IQR) score	44.6 (41.6-47.0)	44.5 (41.3-46.9)	44.5 (41.6-46.9)	44.3 (40.8-46.8)
Self-efficacy, median (IQR)^c^	7.0 (5.2-9.0)	7.0 (5.2-8.9)	7.5 (5.5-9.2)	7.7 (5.5-9.2)
FACT-G7^d^				
No.	447	261	239	205
Median (IQR)	19.0 (14.0-23.0)	19.0 (14.0-23.0)	19.0 (15.0-23.0)	19.0 (15.0-23.0)

Abbreviations: COST, Comprehensive Score for Financial Toxicity; FACT-G7, Functional Assessment of Cancer Therapy–General (rapid version); NDI, Neighborhood Deprivation Index.

^a^ American Indian or Alaska Native, Asian, multiple selected, Native Hawaiian or Other Pacific Islander, not reported, and unknown are the subcategories included in the other category for race.

^b^ Range, 0-100; higher score indicates greater deprivation.

^c^ Range, 1-10; higher score indicates better self-efficacy.

^d^ Range, 0-28; higher score indicates better quality of life.

There was 54% attrition from baseline to 12 months, a common phenomenon in PRO studies.^22,23^ Participants who missed the 12-month survey were more likely to be Black, be Hispanic or Latino, have low education, be unemployed, have Medicaid or be uninsured, exhibit lower baseline self-efficacy, and have a lower FACT-G7 (eTable 1 in Supplement 1).

### Baseline Covariates of Financial Hardship

In univariable analyses, higher baseline COST score (better financial well-being) was significantly associated with older age (β = 0.3; 95% CI, 0.2 to 0.3; *P* < .001), higher FACT-G7 (β = 1.2; 95% CI, 1.0 to 1.3; *P* < .001), higher baseline self-efficacy score (β = 2.2; 95% CI, 1.8 to 2.6; *P* < .001), lower neighborhood deprivation (β = −0.6; 95% CI, −0.9 to −0.3; *P* < .001), and lack of receipt of chemotherapy (β = 5.8; 95% CI, 3.6 to 8.0; *P* < .001). Statistically significant differences in baseline COST were observed by differing levels of household income, cancer stage, cancer type, education, employment, marital status, primary health insurance status, and race (eTable 2 in Supplement 1).

### Financial Hardship

At baseline, the mean (SD) COST score was 23.5 (11.9), and mean (SD) COST was 28.6 (11.7) at 12 months. In the longitudinal mixed model, improvement in COST score was linearly associated with time, meaning that financial burden improved over the study period. There was an improvement in financial hardship from diagnosis to 12 months of 0.3 (95% CI, 0.2 to 0.3) points per month (*P* < .001). Each 1-unit increase in FACT-G7 was associated with an increase of 0.7 (95% CI, 0.5, 0.9) points in COST score (*P* < .001), and each 1-increase in self-efficacy was associated with an increase of 0.6 (95% CI, 0.2 to 1.0) points in COST score (*P* = .006), but each 1-unit increase in NDI was associated with a decrease of 0.3 (95% CI, −0.5 to −0.1) points in COST score (*P* = .009). Statistically significant differences in COST score were observed by changing levels of employment status, cancer type, education, annual household income, and primary insurance status (Table 2). In particular, participants who were employed reported higher COST score over time compared with unemployed participants (β = 2.6; 95% CI, 0.3 to 4.8), participants with some college or more demonstrated higher COST score over time compared with participants with high school education or less (β = 1.8; 95% CI, 0.1 to 3.4), and participants with rectal cancer exhibited lower COST score over time compared with participants with colon cancer (β = −2.8; 95% CI, −4.5 to −1.1) (Table 2).

**Table 2.** Mixed Model and 12-Month Difference Model of COST Outcomes

Covariate	12-mo longitudinal model (n = 447)		12-mo difference model (n = 205)	
	β (95% CI)^a^	*P* value^b^	β (95% CI)^a^	*P* value^b^
Intercept	14.4 (2.2 to 26.6)	.02	5.3 (−10.5 to 21.2)	.51
Time point, mo^c^	0.3 (0.2 to 0.3)	<.001	NA	NA
Age at registration, y	0.0 (−0.1 to 0.1)	.98	0.0 (−0.1 to 0.2)	.74
Baseline COST^c^	NA	NA	−0.4 (−0.6 to −0.3)	<.001
Cancer stage				
Stage II	−2.1 (−4.5 to 0.4)	.21	−0.2 (−3.4 to 2.9)	.98
Stage III	−2.3 (−5.0 to 0.5)		−0.4 (−4.3 to 3.4)	
Stage I	0 [Reference]		0 [Reference]	
Cancer type				
Rectal	−2.8 (−4.5 to −1.1)	.005	−3.1 (−5.4 to −0.7)	.002
Rectosigmoid junction	−0.8 (−4.5 to 2.9)		−8.3 (−13.9 to −2.6)	
Colon	0 [Reference]		0 [Reference]	
Chemotherapy				
No	0.0 (−2.1 to 2.1)	.98	−1.8 (−4.9 to 1.2)	.24
Yes	0 [Reference]		0 [Reference]	
Comorbidities				
1	−1.1 (−3.4 to 1.1)	.17	0.9 (−2.2 to 3.9)	.22
>1	−1.9 (−3.9 to 0.1)		−1.3 (−4.0 to 1.5)	
None	0 [Reference]		0 [Reference]	
Education				
Some college and advanced degree	1.8 (0.1 to 3.4)	.04	0.8 (−1.6 to 3.1)	.78
Not answered	−4.7 (−12.9 to 3.5)		4.1 (−17.9 to 26.0)	
≤High school	0 [Reference]		0 [Reference]	
Employment				
Employed	2.6 (0.3 to 4.8)	<.001	0.2 (−3.2 to 3.6)	.36
Not answered	2.8 (−4.2 to 9.8)		2.5 (−7.0 to 11.9)	
Retired	6.2 (3.5 to 8.9)		2.7 (−1.3 to 6.6)	
Unemployed	0 [Reference]		0 [Reference]	
FACT-G7	0.7 (0.5 to 0.9)	<.001	0.3 (0.0 to 0.6)	.03
Sex				
Female	0.6 (−1.0 to 2.1)	.46	1.6 (−0.5 to 3.7)	.13
Male	0 [Reference]		0 [Reference]	
Income, $				
30 000 to 59 999	1.0 (−1.2 to 3.3)	<.001	0.1 (−3.1 to 3.3)	.31
≥60 000	5.7 (3.4 to 8.1)		1.7 (−1.8 to 5.1)	
Not answered	5.2 (−0.4 to 10.8)		−5.0 (−13.1 to 3.1)	
≤29 999	0 [Reference]		0 [Reference]	
Marital status				
Married, living with partner	0.7 (−1.1 to 2.5)	.69	0.8 (−1.6 to 3.3)	.71
Not answered	3.0 (−8.9 to 14.9)		3.9 (−11.0 to 18.7)	
Unpartnered	0 [Reference]		0 [Reference]	
Primary insurance				
Medicaid, single service, no insurance	2.4 (−1.0 to 5.7)	.01	4.5 (−1.2 to 10.2)	.28
Military, Indian, Medicare	3.3 (1.0 to 5.5)		−0.2 (−3.7 to 3.2)	
Private insurance	0 [Reference]		0 [Reference]	
Race				
Black	−3.5 (−6.6 to −0.5)	.06	−1.1 (−6.2 to 3.9)	.91
Other^d^	−1.1 (−4.0 to 1.7)		−0.3 (−4.9 to 4.4)	
White	0 [Reference]		0 [Reference]	
Safety-net hospital				
No or no answer	−0.4 (−2.6 to 1.9)	.75	1.2 (−1.9 to 4.3)	.46
Yes	0 [Reference]		0 [Reference]	
Self-efficacy^e^	0.6 (0.2 to 1.0)	.006	0.1 (−0.6 to 0.7)	.81
NDI^f^	−0.3 (−0.5 to −0.1)	.009	−0.0 (−0.3 to 0.3)	>.99

Abbreviations: COST, Comprehensive Score for Financial Toxicity; FACT-G7, Functional Assessment of Cancer Therapy–General (rapid version); NA, not applicable; NDI, Neighborhood Deprivation Index.

^a^ Parameter estimates for continuous covariates are interpreted as the change in mean COST score per 1-unit increase in the covariate. Parameter estimates for categorical covariates are interpreted as the difference in mean COST score in comparison with the reference level.

^b^ For categorical variables with more than 2 levels, *P* values are from the overall test of the null hypothesis that all estimates are equal against the alternative that at least 1 is different.

^c^ Baseline COST is only included in the difference model, and time point is only included in the mixed model.

^d^ American Indian or Alaska Native, Asian, multiple selected, Native Hawaiian or Other Pacific Islander, not reported, and unknown are the subcategories included in the ‘Other’ category for Race.

^e^ Higher score indicates greater self-efficacy.

^f^ Higher score indicates greater deprivation.

**Figure 2.**
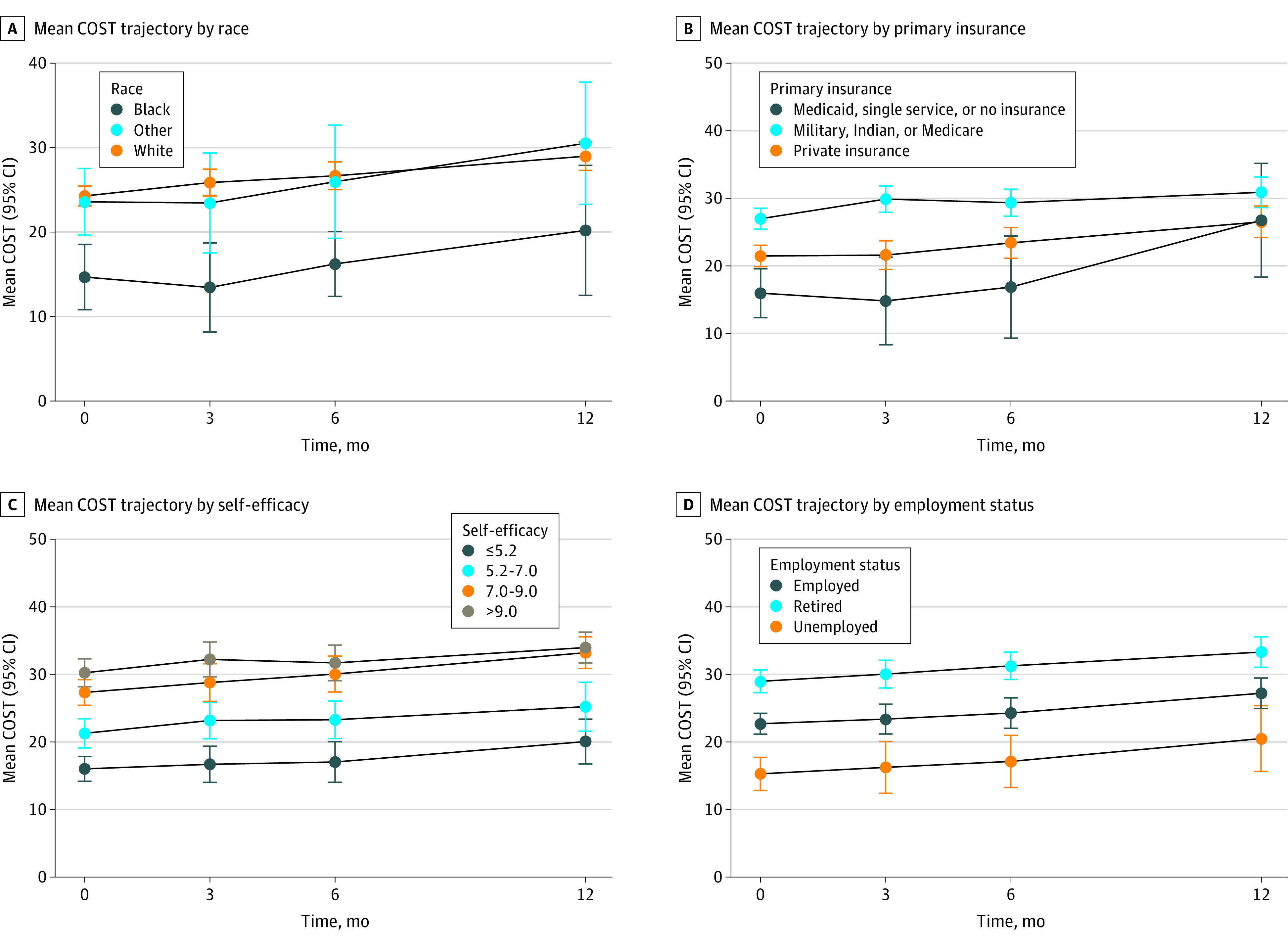
Observed Longitudinal Trajectories of COST Self-efficacy was assessed with the 6-item Stanford Self-Efficacy for Management Chronic Disease, with higher score indicating better self-efficacy.^18^ Other race includes American Indian or Alaska Native, Asian, multiple races, Native Hawaiian or Other Pacific Islander, not reported, and unknown. COST indicates Comprehensive Score for Financial Toxicity, with higher score indicating less financial toxicity.

Ordinary linear regression was also fit to model the change of COST from baseline to the 12-month follow-up (Table 2). This analysis included 205 patients who completed both the baseline and 12-month assessments and with available covariates. In this model, baseline COST, type of cancer, and FACT-G7 were significant covariates (Table 2). The change in COST from baseline to 12 months decreased 0.4 (95% CI, −0.6 to −0.3) points for each 1-unit increase in COST at baseline, meaning participants who had more financial worry at baseline were the ones who were more likely to improve (Table 2). Compared with participants with colon cancer, participants with rectal or rectosigmoid tumors were less likely to experience an improvement in financial hardship from baseline to 12 months (Table 2). When we assessed the pattern of the observed COST was displayed by race, primary insurance, baseline self- efficacy, and employment status, all groups had an improved COST score over time; however Black participants; participants with Medicaid, single service or no insurance; participants with low self-efficacy scores, and unemployed participants did not have the same degree of improvement as their counterpart groups, which were confirmed by our interaction analysis (Figure 2).

To assess the influence from attrition over time, 2 additional longitudinal models were performed to assess whether the covariates were similar at each time point. The 2 additional models included a model with baseline and 3- and 6-month follow-ups and a model with baseline and 3-month follow-up. Similar variables (cancer type, education, employment, FACT-G7, income, insurance, self-efficacy, NDI, and time) were significant in all 3 models. (eTable 3 in Supplement 1) Furthermore, the sensitivity analysis following multiple imputation indicated that while the estimates of regression coefficients were altered, the significant covariates remained largely unchanged. The only difference was a change in the overall *P* value for race from not significant to significant (eTable 5 in Supplement 1)

## Discussion

In this prospective cohort study of patients with CRC treated with curative intent in community settings, patient-reported psychological financial toxic effects decreased over the course of a year. Factors associated with promoting financial well-being included individual-level factors of having higher income, having higher education, having government insurance (compared with private insurance), and being employed. These findings echo previous cross-sectional^3,4,24^ and longitudinal studies^25,26,27^ using the COST or other measure of financial hardship.^3,4^ At the neighborhood-level, NDI had a negative association with financial well-being even after adjusting for individual characteristics. A study by Sadigh et al^12^ found similar associations of NDI, with individuals residing in the highest deprived neighborhoods having a higher probability of endocrine therapy discontinuation in a breast cancer population. While most individual and neighborhood-level covariates of financial hardship are not modifiable by practice-delivered interventions, we found that higher self-efficacy across all levels of NDI was associated with less financial hardship and may represent an intervention target. Low self-efficacy has been similarly found to be associated with financial hardship in adolescent and adult cancer populations,^28,29,30^ as well as in other chronic diseases.^31^

In the US, most individuals are more worried about the financial hardship of a major illness than the illness itself.^32^ Among individuals diagnosed with cancer, and potentially prior to actual receipt of cancer treatment-related medical bills, financial hardship may be driven by the anticipation and worry of the inability to pay. By 12 months after diagnosis, patients had navigated using their health insurance plans and coping with direct and indirect costs, which may ease financial anxiety related to uncertainty. This premise is supported by our data showing that most patients with early-stage cancer had financial hardship at baseline and those who were best equipped to cope with actual costs (eg, higher income) experienced more improvement. A study by Friedes et al^27^ reported similar findings of improved financial well-being in the first 6 months of a lung cancer diagnosis. This finding is likely only true for early-stage disease, as patients with metastatic disease will have a prolonged time of dealing with cancer care costs.

We used the COST instrument, one of the most rigorously developed and frequently used measures of financial burden.^11^ It is important to highlight that this instrument predominantly measures the psychological impacts of financial hardship. Reassuringly, while individuals with government insurance (including Medicaid) reported worse financial hardship at baseline compared with those with other forms of insurance, little difference in residual financial hardship was observed at the end of the study period across these insurance groups. Nevertheless, notable disparities were observed. Specifically, unemployed individuals and those with low self-efficacy were more likely to report worse financial hardship at study entry. Moreover, while the trajectory of financial hardship among unemployed participants mirrored the larger population, financial hardship was demonstrably worse at every time point assessed, with residual mild financial hardship persisting at the end of the observation period compared with the remainder of the population. A similar pattern was seen with self-efficacy, and further research should explore whether this represents an opportunity for intervention.

Our longitudinal analyses highlight the dynamic nature of financial hardship and critical need to identify interventions to meet the unique needs of patients at different points in the cancer continuum. Individuals with moderate financial hardship at treatment onset, particularly when coupled with unemployment or low self-efficacy, may be ideal candidates for early delivery of supportive care, such as financial navigation and social work assistance, delivered contemporaneous with treatment planning. Even patients equipped to afford costly care may benefit from counseling to mitigate anxiety related to the perception of downstream financial hardship. For all patients, improving self-efficacy through targeted education may be a modifiable factor in improving financial hardship. Future analysis of our data will assess the associations of the other domains of material and coping behaviors. Significant psychological financial hardship at treatment onset or residual financial hardship after treatment completion may indicate coexistence or future development of material hardship, such as the use of debt to manage care costs, or maladaptive coping mechanisms, such as foregoing necessary care or medication.

Our results contrast with literature on patients with metastatic disease, highlighting the asymmetry of financial hardship experiences. A study by Shankaran et al^33^ reported that cumulative incidence of major financial hardship was observed in 71.3% of patients with metastatic CRC at 12 months after diagnosis. The analysis by Shankaran et al^33^ primarily measured material financial hardship and is difficult to compare with our primary analysis, which focused on psychological outcomes. In addition, there may be differences in the financial hardship experienced by patients with metastatic disease who potentially have more prolonged direct and indirect costs. In this population, material hardship may be less responsive over time, even if psychological financial hardship is dynamic. Additional studies are needed with consistent measurement across studies.

### Limitations

This study has several limitations. Response rates declined over time, particularly among traditionally marginalized populations (eg, individuals with low socioeconomic status or who self-identify as Black or Hispanic or Latino), the same populations posited as most at risk for financial hardship. We may have underestimated the between-group differences, and the upward financial hardship trajectory may be muted in these groups. Our sample included a predominantly White population with a higher income. Collection of other individual-level factors associated with financial health (eg, presence of a savings account) helped describe the population with more granularity but generalizability may be limited in the overall of early-stage CRC population, which is more economically and racially diverse. Because the enrollment criteria allowed for participants to be up to 60 days from diagnosis, some patients had already had a surgical procedure; therefore, the baseline may not reflect their financial status at diagnosis. Like most studies, we do not have data on patients who did not choose to enroll in this study; therefore, selection bias may be present. This bias was not evident when we compared the limited collected data. Despite these limitations, we believe that this national sample of patients with newly diagnosed CRC treated with curative intent at community oncology sites across the US is informative, representing a critical companion to the emerging financial hardship data in the metastatic setting.

## Conclusions

In this cohort study among patients with newly diagnosed, nonmetastatic CRC, financial hardship was common at diagnosis, regardless of sociodemographic characteristics. Reassuringly, among the patients in this study treated with curative intent, the trajectory within the psychological domain was one of increasing financial well-being over time. Despite the general upward trajectory, the magnitude of financial hardship and the presence of residual financial hardship after treatment completion varied by sociodemographic factors. Interventions to mitigate financial hardship have included financial screening for financial hardship, navigation and education programs,^34,35,36^ referral order sets integrated into electronic medical record,^37^ and the establishment of multidisciplinary financial tumor boards,^38^ which have demonstrated feasibility and promising patient-level outcomes. Nevertheless, there is a need for tailored interventions to address the unique needs of specific patient groups. The findings of this study of a relatively homogenous patient population with early-stage disease suggest that interventions focused on screening, reassurance, and supporting self-efficacy, along with financial navigation, should be delivered in tandem with treatment planning. While financial counseling and navigation of direct and indirect treatment-related costs should be available for all, where capacity for these services is limited, patient- and neighborhood-level sociodemographic characteristics may help prioritize service delivery. The impact of financial navigation interventions remains to be tested.

## Notes

Supplement 1. **eTable 1.** Covariates of Participants Missing COST at 12 Months **eTable 2.** Univariable Analyses Between Baseline COST and Covariates **eTable 3.** Comparison of 3-, 6-, and 12-Month Longitudinal Models Results **eTable 4.** Participant Characteristics, by Cohort **eTable 5.** Comparisons After Multiple Imputation

Supplement 2.
Data Sharing Statement
