# Supplementary material for: Patient-Reported Financial Burden of Treatment for Colon or Rectal Cancer
Source: JAMA Netw Open. 2024 Jan 9;7(1):e2350844. doi: 10.1001/jamanetworkopen.2023.50844 (PMC10777253; doi:10.1001/jamanetworkopen.2023.50844)
Supplement: Supplement 1. — eTable 1. Covariates of Participants Missing COST at 12 Months eTable 2. Univariable Analyses Between Baseline COST and Covariates eTable 3. Comparison of 3-, 6-, and 12-Month Longitudinal Models Results eTable 4. Participant Characteristics, by Cohort eTable 5. Comparisons After Multiple Imputation [file jamanetwopen-e2350844-s001.pdf]

Supplemental Online Content

Kircher S, Duan F, An N, et al. Patient-reported financial burden of treatment for colon or rectal cancer. *JAMA Netw Open*. 2024;7(1):e2350844. doi:10.1001/jamanetworkopen.2023.50844

eTable 1. Covariates of Participants Missing COST at 12 Months

eTable 2. Univariable Analyses Between Baseline COST and Covariates

eTable 3. Comparison of 3-, 6-, and 12-Month Longitudinal Models Results

eTable 4. Participant Characteristics, by Cohort

eTable 5. Comparisons After Multiple Imputation

This supplemental material has been provided by the authors to give readers additional information about their work.

© 2023 Kircher S et al. *JAMA Network Open.*

eTable 1. Covariates of Participants Missing COST at 12-months

Characteristic	12 Month COST Completed (N=207)	12 Month COST Not Completed (N=243)	P-value [a]
**Age (Years)**			
n	207	243	0.10
Mean (SD)	62.0 (12.1)	60.1 (12.0)	
**Gender, n (%)**			
Female	99 (47.8)	111 (45.7)	0.65
Male	108 (52.2)	132 (54.3)	
**Race, n (%)**			
Black	10 (4.8)	23 (9.5)	0.01*
White	186 (89.9)	193 (79.4)	
Other [b]	11 (5.3)	27 (11.1)	
**Ethnicity, n (%)**			
Hispanic or Latino	2 (1.0)	12 (4.9)	0.02*
Not Hispanic or Latino	198 (95.7)	226 (93.0)	
Not Reported [c]	3 (1.4)	1 (0.4)	
Unknown [c]	4 (1.9)	4 (1.6)	
**Education, n (%)**			
High School or Less	69 (33.3)	109 (44.9)	0.009**
Some College and Advanced Degree	137 (66.2)	130 (53.5)	
Not Answered [c]	1 (0.5)	4 (1.6)	
**Marital Status, n (%)**			
Married, Living with partner	134 (64.7)	161 (66.3)	0.84
Unpartnered	71 (34.3)	82 (33.7)	
Not Answered [c]	2 (1.0)	0 (0.0)	
**Annual Household Income, n (%)**			
Up to $29,999	45 (21.7)	66 (27.2)	0.05
$30,000 to $59,999	56 (27.1)	80 (32.9)	
$60,000 and greater	101 (48.8)	91 (37.4)	
Not Answered [c]	5 (2.4)	6 (2.5)	
**Employment, n (%)**			
Employed	101 (48.8)	113 (46.5)	0.03*
Retired	78 (37.7)	75 (30.9)	
Unemployed	25 (12.1)	52 (21.4)	
Not Answered [c]	3 (1.4)	3 (1.2)	
**Safety-net Hospital, n (%)**			
No/Not Answered	177 (85.5)	207 (85.2)	0.92
Yes	30 (14.5)	36 (14.8)	
**Chemotherapy, n (%)**			

© 2023 Kircher S et al. *JAMA Network Open.*

No	94 (45.4)	89 (36.6)	0.06
Yes	113 (54.6)	154 (63.4)	
**Primary Health Insurance Provider, n (%)**			0.04*
Medicaid, Single service, No insurance	8 (3.9)	22 (9.1)	
Military, Indian, Medicare	100 (48.3)	97 (39.9)	
Private insurance	99 (47.8)	124 (51.0)	
**Cancer Type, n (%)**			0.69
Colon Cancer	132 (63.8)	157 (64.6)	
Rectal Cancer	67 (32.4)	73 (30.0)	
Rectosigmoid Junction	8 (3.9)	13 (5.3)	
**Cancer Stage, n (%)**			0.06
Stage l	39 (18.8)	29 (11.9)	
Stage ll	67 (32.4)	73 (30.0)	
Stage lll	101 (48.8)	141 (58.0)	
**Comorbidities, n (%)**			0.47
1	56 (27.1)	55 (22.6)	
>1	104 (50.2)	135 (55.6)	
None	47 (22.7)	53 (21.8)	
**NDI (Higher score means greater neighborhood deprivation)**			0.13
n	207	243	
Median (Q1, Q3)	44.3 (40.8, 46.8)	45.0 (42.2, 47.2)	
**Baseline Self-efficacy**			0.01*
n	207	243	
Median (Q1, Q3)	7.7 (5.5, 9.2)	6.7 (4.8, 8.7)	
**Baseline FACT-G7**			0.01*
n	205	242	
Median (Q1, Q3)	19.0 (15.0, 23.0)	18.0 (13.0, 22.0)	

Note:
N=Number of participants; SD=Standard Deviation; Q1=Lower quartile; Q3=Upper quartile.
Mean and standard deviation were summarized for symmetric variables, median and quartile range for asymmetric continuous variables, and frequencies and percentages for categorical variables.
[a] P-values compare participants with 12 months in the analysis set versus without 12 months. For continuous variables, the p-value corresponds to the t-test or the non-parametric Wilcoxon rank sum test, as appropriate. For categorical variables, the p-value corresponds to the chi-square test or the exact version of chi-square test, as appropriate.
[b] American Indian or Alaska Native, Asian, Multiple selected, Native Hawaiian or Other Pacific Islander, Not Reported, and Unknown are the subcategories included in the 'Other' category for Race.
[c] The p-value for the comparison was performed after removing these categories.
Percentages shown are column based and the denominator is the total N in column including the not answered category.
***<0.05 ** <0.01 *** <0.001**

© 2023 Kircher S et al. *JAMA Network Open.*

eTable 2. Univariable Analyses between Baseline COST and Covariates

Covariate	Category or Units	Estimate (95% CI) [a]	P-value [b]
Age		0.3 (0.2, 0.3)	<.001***
Annual Household Income	$30,000 to $59,999	4.9 (2.1, 7.7)	<.001***
	$60,000 and greater	9.5 (6.8, 12.1)	
	Not Answered	9.8 (2.8, 16.8)	
	Up to $29,999+		
Cancer Stage	Stage ll	-5.7 (-9.1, -2.4)	<.001***
	Stage lll	-8.4 (-11.5, -5.3)	
	Stage l+		
Cancer Type	Rectal Cancer	-3.8 (-6.1, -1.4)	0.008**
	Rectosigmoid Junction	-0.3 (-5.5, 4.9)	
	Colon Cancer+		
Chemotherapy	No	5.8 (3.6, 8)	<.001***
	Yes+		
Comorbidities	1	-2.2 (-5.4, 1.0)	0.20
	>1	-2.5 (-5.2, 0.3)	
	None+		
Education	Some College and Advanced Degree	4.2 (2.0, 6.5)	<.001***
	Not Answered	0.6 (-9.8, 11.0)	
	High School or Less +		
Employment	Employed	7.4 (4.6, 10.3)	<.001***
	Retired	4.0 (-5.0, 13.1)	
	Not Answered	13.7 (10.7, 16.7)	
	Unemployed+		
FACT-G7		1.2 (1.0, 1.3)	<.001***
Gender	Female	0.1 (-2.1, 2.3)	0.91
	Male+		
Marital Status	Married, Living with partner	4.2 (1.9, 6.5)	0.002**
	Not Answered	2.3 (-14.1, 18.6)	
	Unpartnered+		
Primary Health Insurance Provider	Medicaid, Single service, No insurance	-5.5 (-9.9, -1.1)	<.001***
	Military, Indian, Medicare	5.5 (3.3, 7.7)	
	Private insurance+		
Race	Black	-9.6 (-13.7, -5.5)	

© 2023 Kircher S et al. *JAMA Network Open.*

	Other [c]	-0.7 (-4.6, 3.2)	<.001***
	White+		
Safety-net Hospital	No/Unknown	0.9 (-2.2, 4.0)	0.57
	Yes+		
Self-efficacy		2.2 (1.8, 2.6)	<.001***
NDI (Higher score means greater neighborhood deprivation)		-0.6 (-0.9, -0.3)	<.001***
NDI Quartile	Quartile 1, lowest deprivation (<=41.9)	5.8 (2.8, 8.8)	0.001**
	Quartile 2 (41.9-44.5]	3.8 (0.6, 6.9)	
	Quartile 3 (44.5-47.0]	1.8 (-1.2, 4.8)	
	Quartile 4, highest deprivation (>47.0) +		

Note:
[a] Parameter estimates for continuous covariates are interpreted as the change in mean baseline COST score per unit increase in the covariate. Parameter estimates for categorical covariates are interpreted as the difference in mean baseline COST score in comparison with the reference level.
[b] P value: For the categorical variable with more than 2 levels, this is the p value from the overall test of the null hypothesis that all estimates are equal against the alternative that at least one is different.
[c] American Indian or Alaska Native, Asian, Multiple selected, Native Hawaiian or Other Pacific Islander, Not Reported, and Unknown are the subcategories included in the 'Other' category for Race.
+Reference group
***<0.05 ** <0.01 *** <0.001**

© 2023 Kircher S et al. *JAMA Network Open.*

eTable 3. Comparison of 3, 6, and 12-month Longitudinal Models Results

	**12 Month Mixed Model [a] (N=447) [d]**	**6 Month Mixed Model [b] (N=447) [d]**	**3 Month Mixed Model [c] (N=447) [d]**
**Covariate**	**P-value**	**P-value**	**P-value**
Intercept	0.02	0.01	0.05
Age at Registration	0.98	>0.99	0.99
Cancer Stage	0.21	0.22	0.10
Cancer Type	0.005**	0.008**	0.02*
Chemotherapy	0.98	0.74	0.93
Comorbidities	0.17	0.19	0.31
Education	0.04*	0.04*	0.03*
Employment	<.001***	<.001***	0.002**
FACT-G7	<.001***	<.001***	<.001***
Gender	0.46	0.61	0.64
Income	<.001**	<.001**	<.001**
Marital status	0.69	0.79	0.89
Primary Insurance	0.01*	0.01*	0.006**
Race	0.06	0.07	0.05 [e] *
Safety-net Hospital	0.75	0.57	0.96
Self-efficacy	0.006**	0.005**	0.005**
NDI (Higher score means greater neighborhood deprivation)	0.009**	0.005**	0.02*
Time	<.001***	0.05 [e] *	0.10

Note:
[a] Longitudinal multivariable model including timepoints baseline, 3-month, 6-month, and 12-month.
[b] Longitudinal multivariable model including timepoints baseline, 3-month, 6-month.
[c] Longitudinal multivariable model including timepoints baseline, 3-month.
[d] There are 447 participants with baseline COST measures and available covariates included in the longitudinal mixed model.
[e] Borderline significant for 6 month mixed model and 3 month mixed model are 0.0474 and 0.0472.
***<0.05 ** <0.01 *** <0.001**

© 2023 Kircher S et al. *JAMA Network Open.*

eTable 4. Participant Characteristics, by Cohort

Baseline Characteristics	Registered Participants (N=565)	In Analysis Participants (N=450)
**Age (Years)**		
n	565	450
Mean (SD)	61.2 (12.1)	61.0 (12.0)
**Gender, n (%)**		
Male	308 (54.5)	240 (53.3)
Female	257 (45.5)	210 (46.7)
**Race, n (%)**		
Black	44 (7.8)	33 (7.3)
White	474 (83.9)	379 (84.2)
Other [a]	47 (8.3)	38 (8.4)
**Ethnicity, n (%)**		
Hispanic or Latino	16 (2.8)	14 (3.1)
Not Hispanic or Latino	532 (94.2)	424 (94.2)
Not Reported	6 (1.1)	4 (0.9)
Unknown	11 (1.9)	8 (1.8)
**Chemo, n (%)**		
Yes	324 (57.3)	267 (59.3)
No	241 (42.7)	183 (40.7)
**Primary health insurance provider, n (%)**		
Private insurance	283 (50.1)	223 (49.6)
Military, Indian, Medicare	238 (42.1)	197 (43.8)
Medicaid, Single service, No insurance	44 (7.8)	30 (6.7)
**Cancer Type, n (%)**		
Colon Cancer	350 (61.9)	289 (64.2)
Rectal Cancer	187 (33.1)	140 (31.1)
Rectosigmoid Junction	28 (5.0)	21 (4.7)
**Cancer Stage, n (%)**		
Stage l	87 (15.4)	68 (15.1)
Stage ll	179 (31.7)	140 (31.1)
Stage lll	299 (52.9)	242 (53.8)
**NDI (Higher score means greater neighborhood deprivation), n (%)**		
Quartile 1, lowest deprivation (<=41.9)	135 (23.9)	117 (26.0)
Quartile 2 (41.9-44.5]	140 (24.8)	99 (22.0)
Quartile 3 (44.5-47.0]	147 (26.0)	122 (27.1)
Quartile 4, highest deprivation (>47.0)	143 (25.3)	112 (24.9)
n	565	450
Median (Q1, Q3)	44.5 (41.9,47.0)	44.6 (41.6,47.0)

Note: N=Number of participants; n=Number of participants in analysis; SD=Standard Deviation; Q1=Lower quartile; Q3=Upper quartile. Mean and standard deviation were summarized for symmetric variables, median and quartile range for asymmetric continuous variables, and frequencies and percentages for categorical variables.
Percentages shown are column based and the denominator is the total N in column including the not answered category.
Participants in analysis are those participants with available baseline COST.

[a] American Indian or Alaska Native, Asian, Multiple selected, Native Hawaiian or Other Pacific Islander, Not Reported, and Unknown are the subcategories included in the 'Other' category for Race.

© 2023 Kircher S et al. *JAMA Network Open.*

eTable 5. Comparisons after Multiple Imputation

A) 12-Month Longitudinal Model

		12 Month Longitudinal Model (N=447) [d]		Multiple Imputation Model (N=501) [g]	
**Covariate**	**Category or Units**	**Estimate (95% CI) [c]**	**P-value [a]**	**Estimate (95% CI)**	**P-value [a]**
Intercept		14.4 (2.2, 26.6)	0.02*	17.7 (6.8, 28.5)	0.002**
Time Point [b]	Months	0.3 (0.2, 0.3)	<.001***	0.3 (0.3, 0.4)	<.001***
Age at Registration	Years	0.0 (-0.1, 0.1)	0.98	0.0 (-0.1, 0.1)	0.67
Baseline COST [b]		N/A		N/A	
Cancer Stage	Stage ll	-2.1 (-4.5, 0.4)	0.21	-1.2 (-3.3, 0.9)	0.54
	Stage lll	-2.3 (-5.0, 0.5)		-1.0 (-3.4, 1.4)	
	Stage l+				
Cancer Type	Rectal Cancer	-2.8 (-4.5, -1.1)	0.005**	-2.2 (-3.6, -0.7)	0.02*
	Rectosigmoid Junction	-0.8 (-4.5, 2.9)		-0.1 (-3.3, 3.0)	
	Colon Cancer+				
Chemotherapy	No	0.0 (-2.1, 2.1)	0.98	0.3 (-1.5, 2.1)	0.74
	Yes+				
Comorbidities	1	-1.1 (-3.4, 1.1)	0.17	-1.0 (-3.1, 1.0)	0.24
	>1	-1.9 (-3.9, 0.1)		-1.6 (-3.4, 0.3)	
	None+				
Education	Some College and Advanced Degree	1.8 (0.1, 3.4)	0.04*	1.9 (0.3, 3.4)	0.01*
	Not Answered	-4.7 (-12.9, 3.5)		-5.3 (-12.6, 2.0)	
	High School or Less+				
Employment	Employed	2.6 (0.3, 4.8)	<.001***	2.3 (0.2, 4.4)	<.001***
	Not Answered	2.8 (-4.2, 9.8)		2.2 (-3.4, 7.9)	
	Retired	6.2 (3.5, 8.9)		6.2 (3.8, 8.6)	
	Unemployed+				
FACT-G7		0.7 (0.5, 0.9)	<.001***	0.7 (0.5, 0.8)	<.001***
Gender	Female	0.6 (-1.0, 2.1)	0.46	0.5 (-0.8, 1.9)	0.46
	Male+				
Income	$30,000 to $59,999	1.0 (-1.2, 3.3)	<.001***	0.3 (-1.8, 2.3)	<.001***
	$60,000 and greater	5.7 (3.4, 8.1)		4.7 (2.6, 6.9)	
	Not Answered	5.2 (-0.4, 10.8)		4.9 (0.0, 9.8)	
	Up to $29,999+				
Marital Status	Married, Living with partner	0.7 (-1.1, 2.5)	0.69	0.7 (-1.0, 2.3)	0.70
	Not Answered	3.0 (-8.9, 14.9)		-1.0 (-9.2, 7.2)	
	Unpartnered+				
Primary Insurance	Medicaid, Single service, No insurance	2.4 (-1.0, 5.7)	0.01*	0.9 (-1.9, 3.7)	0.001**
	Military, Indian, Medicare	3.3 (1.0, 5.5)		3.7 (1.7, 5.6)	

© 2023 Kircher S et al. *JAMA Network Open.*

	Private insurance[+]				
Race	Black	-3.5 (-6.6, -0.5)	0.06	-3.7 (-6.3, -1.1)	0.01*
	Other [f]	-1.1 (-4.0, 1.7)		-1.5 (-4.0, 1.0)	
	White[+]				
Safety-net Hospital	No/Not Answered	-0.4 (-2.6, 1.9)	0.75	-0.2 (-2.2, 1.8)	0.84
	Yes[+]				
Self-efficacy	Higher score means more confidence	0.6 (0.2, 1.0)	0.006**	0.6 (0.2, 1.0)	0.001**
NDI	Higher score means greater neighborhood deprivation	-0.3 (-0.5, -0.1)	0.009**	-0.3 (-0.5, -0.2)	<.001***

B) 12-Month Difference Model

		**12 Month Difference Model (N=205) [e]**		**Multiple Imputation Model (N=501) [g]**	
**Covariate**	**Category or Units**	**Estimate (95% CI)**	**P-value [a]**	**Estimate (95% CI)**	**P-value [a]**
Intercept		5.3 (-10.5, 21.2)	0.51	15.4 (4.3, 26.5)	0.006**
Time Point [b]	Months	N/A		N/A	
Age at Registration	Years	0.0 (-0.1, 0.2)	0.74	0.0 (-0.1, 0.1)	0.43
Baseline COST [b]		-0.4 (-0.6, -0.3)	<.001***	-0.5 (-0.5, -0.4)	<.001***
Cancer Stage	Stage ll	-0.2 (-3.4, 2.9)	0.98	0.8 (-1.3, 3.0)	0.73
	Stage lll	-0.4 (-4.3, 3.4)		0.5 (-1.9, 3.0)	
	Stage l[+]				
Cancer Type	Rectal Cancer	-3.1 (-5.4, -0.7)	0.002**	-1.4 (-2.9, 0.1)	0.01*
	Rectosigmoid Junction	-8.3 (-13.9, -2.6)		-3.9 (-7.1, -0.8)	
	Colon Cancer[+]				
Chemotherapy	No	-1.8 (-4.9, 1.2)	0.24	-0.1 (-2.0, 1.7)	0.83
	Yes[+]				
Comorbidities	1	0.9 (-2.2, 3.9)	0.22	0.6 (-1.5, 2.7)	0.38
	>1	-1.3 (-4.0, 1.5)		-0.6 (-2.5, 1.2)	
	None[+]				
Education	Some College and Advanced Degree	0.8 (-1.6, 3.1)	0.78	0.7 (-0.9, 2.2)	0.41
	Not Answered	4.1 (-17.9, 26.0)		-0.2 (-7.6, 7.2)	
	High School or Less[+]				
Employment	Employed	0.2 (-3.2, 3.6)	0.36	0.6 (-1.5, 2.7)	0.15
	Not Answered	2.5 (-7.0, 11.9)		2.2 (-3.6, 7.9)	
	Retired	2.7 (-1.3, 6.6)		2.3 (-0.1, 4.8)	
	Unemployed[+]				
FACT-G7		0.3 (0.0, 0.6)	0.03*	0.2 (0.1, 0.4)	<.001***
Gender	Female	1.6 (-0.5, 3.7)	0.13	0.6 (-0.8, 2.0)	0.43
	Male[+]				
Income	$30,000 to $59,999	0.1 (-3.1, 3.3)	0.31	-1.2 (-3.3, 0.9)	0.77

© 2023 Kircher S et al. *JAMA Network Open.*

	$60,000 and greater	1.7 (-1.8, 5.1)		-0.9 (-3.1, 1.4)	
	Not Answered	-5.0 (-13.1, 3.1)		-1.7 (-6.7, 3.3)	
	Up to $29,999[+]				
Marital Status	Married, Living with partner	0.8 (-1.6, 3.3)	0.71	0.1 (-1.6, 1.8)	0.94
	Not Answered	3.9 (-11.0, 18.7)		-1.3 (-9.6, 7.0)	
	Unpartnered[+]				
Primary Insurance	Medicaid, Single service, No insurance	4.5 (-1.2, 10.2)	0.28	-2.3 (-5.1, 0.6)	0.07
	Military, Indian, Medicare	-0.2 (-3.7, 3.2)		1.2 (-0.8, 3.2)	
	Private insurance[+]				
Race	Black	-1.1 (-6.2, 3.9)	0.91	-1.1 (-3.7, 1.6)	0.58
	Other [f]	-0.3 (-4.9, 4.4)		-0.8 (-3.3, 1.8)	
	White[+]				
Safety-net Hospital	No/Not Answered	1.2 (-1.9, 4.3)	0.46	-0.1 (-2.2, 1.9)	0.90
	Yes[+]				
Self-efficacy	Higher score means more confidence	0.1 (-0.6, 0.7)	0.81	0.1 (-0.2, 0.5)	0.41
NDI	Higher score means greater neighborhood deprivation	-0.0 (-0.3, 0.3)	>0.99	-0.1 (-0.3, 0.1)	0.18

Note:
[a] P value: For the categorical variable with more than 2 levels, this is the p value from the overall test of the null hypothesis that all estimates are equal against the alternative that at least one is different.
[b] Baseline cost is only included in the difference model and time point is only included in the mixed model
[c] Parameter estimates for continuous covariates are interpreted as the change in mean COST score per unit increase in the covariate. Parameter estimates for categorical covariates are interpreted as the difference in mean COST score in comparison with the reference level.
[d] There are 447 participants with baseline COST measures and available covariates included in the longitudinal mixed model.
[e] There are 205 participants with both baseline and 12-month COST measures and available covariates included in the difference model.
[f] American Indian or Alaska Native, Asian, Multiple selected, Native Hawaiian or Other Pacific Islander, Not Reported, and Unknown are the subcategories included in the 'Other' category for Race.
[g] There are 501 participants included in the multiple imputation model. Among these participants, 38 cases have missing PRO data at all timepoints but have data available for their covariates. Although these 38 cases were initially not included in the Consort diagram as participants who did not start the study, we will include them here.
[+]Reference group
***<0.05 ** <0.01 *** <0.001**

© 2023 Kircher S et al. *JAMA Network Open.*
