# Supplementary material for: Patient-Reported Financial Burden of Treatment for Colon or Rectal Cancer
Source: JAMA Netw Open. 2024 Jan 9;7(1):e2350844. doi: 10.1001/jamanetworkopen.2023.50844 (PMC10777253; doi:10.1001/jamanetworkopen.2023.50844)
Supplement: Supplement 2. — Data Sharing Statement [file jamanetwopen-e2350844-s002.pdf]

Data Sharing Statement

Kircher. Patient-Reported Financial Burden of Treatment for Colon or Rectal Cancer. *JAMA Netw Open*. Published January 09, 2024. doi:10.1001/jamanetworkopen.2023.50844

Data
Data available: Yes
Data types: Deidentified participant data
How to access data: Data request would be sent to ECOG ACRIN communications@ecog-acrin.org or the PI at sheetal.kircher@nm.org
When available: With publication

Supporting Documents
Document types: None

Additional Information
Who can access the data: Researchers whose proposed use of the data has been approved via ECOG ACRIN policy.
Types of analyses: For research related projects
Mechanisms of data availability: After approval of a proposal by ECOG ACRIN and signed EA data access agreement.
